# Investigating risk factors for psychological morbidity three months after intensive care: a prospective cohort study

**DOI:** 10.1186/cc11677

**Published:** 2012-10-15

**Authors:** Dorothy M Wade, David C Howell, John A Weinman, Rebecca J Hardy, Michael G Mythen, Chris R Brewin, Susana Borja-Boluda, Claire F Matejowsky, Rosalind A Raine

**Affiliations:** 1 Department of Applied Health Research, University College London (UCL), 1-19 Torrington Place, London, WC1E 7HB, UK; 2 Critical Care Unit, University College London Hospitals NHS Foundation Trust (UCLH), 235 Euston Rd, London, NW1 2BU, UK; 3 Psychology Department, Institute of Psychiatry, Kings College London, Guy's Campus, St. Thomas St, London, SE1 9RT, UK; 4 MRC Unit for Lifelong Health and Ageing, University College London, 33 Bedford Place, London, WC1B 5JU, UK; 5 UCLH/UCL NIHR Biomedical Research Centre, Maple House, 149 Tottenham Court Rd, London W1T 7DN, UK; 6 Psychology Department, University College London, Gower St, London, WC1E 6BT, UK

## Abstract

**Introduction:**

There is growing evidence of poor mental health and quality of life among survivors of intensive care. However, it is not yet clear to what extent the trauma of life-threatening illness, associated drugs and treatments, or patients' psychological reactions during intensive care contribute to poor psychosocial outcomes. Our aim was to investigate the relative contributions of a broader set of risk factors and outcomes than had previously been considered in a single study.

**Methods:**

A prospective cohort study of 157 mixed-diagnosis highest acuity patients was conducted in a large general intensive care unit (ICU). Data on four groups of risk factors (clinical, acute psychological, socio-demographic and chronic health) were collected during ICU admissions. Post-traumatic stress disorder (PTSD), depression, anxiety and quality of life were assessed using validated questionnaires at three months (n =100). Multivariable analysis was used.

**Results:**

At follow-up, 55% of patients had psychological morbidity: 27.1% (95% CI: 18.3%, 35.9%) had probable PTSD; 46.3% (95% CI: 36.5%, 56.1%) probable depression, and 44.4% (95% CI: 34.6%, 54.2%) anxiety. The strongest clinical risk factor for PTSD was longer duration of sedation (regression coefficient = 0.69 points (95% CI: 0.12, 1.27) per day, scale = 0 to 51). There was a strong association between depression at three months and receiving benzodiazepines in the ICU (mean difference between groups = 6.73 points (95% CI: 1.42, 12.06), scale = 0 to 60). Use of inotropes or vasopressors was correlated with anxiety, and corticosteroids with better physical quality of life.

The effects of these clinical risk factors on outcomes were mediated (partially explained) by acute psychological reactions in the ICU. In fully adjusted models, the strongest independent risk factors for PTSD were mood in ICU, intrusive memories in ICU and psychological history. ICU mood, psychological history and socio-economic position were the strongest risk factors for depression.

**Conclusions:**

Strikingly high rates of psychological morbidity were found in this cohort of intensive care survivors. The study's key finding was that acute psychological reactions in the ICU were the strongest modifiable risk factors for developing mental illness in the future. The observation that use of different ICU drugs correlated with different psychological outcomes merits further investigation. These findings suggest that psychological interventions, along with pharmacological modifications, could help reduce poor outcomes, including PTSD, after intensive care.

## Introduction

The mental health of intensive care survivors may be poor. Patients may suffer from post-traumatic stress disorder (PTSD), depression or anxiety with poor quality of life in the months following intensive care [1-3]. It is not clear whether poor psychological outcomes are associated with the traumatic effects of critical illness, intensive care treatment and drugs (clinical risk factors), or mood and stress reactions in intensive care (acute psychological factors). Outcomes might be better explained by chronic physical conditions and psychological history (chronic health factors) or socio-demographic factors, such as low socio-economic position [4]. There is an urgent need to explore the relative effects of a broader set of risk factors than has previously been investigated on different psychosocial outcomes, within a fully-powered single study of mixed-diagnosis, general intensive care patients.

Psychological outcomes after intensive care include PTSD, an "anxiety disorder that often follows exposure to an extreme stressor that causes injury, threatens life or physical integrity" [5]. The person's immediate response involves intense fear, helplessness or horror. The disorder is characterised by three clusters of symptoms: re-experiencing, avoidance and hyper-arousal, that persist for more than a month and cause distress or impaired functioning. Another outcome of interest, depression, is characterised by low mood or loss of interest for more than two weeks, with a range of other symptoms. Anxiety is a normal emotion that may become persistent and inappropriate. In systematic reviews, the median point prevalence of PTSD among intensive care survivors was 22% [1] with 28% prevalence of depression [2]. Rates of anxiety after intensive care vary from 5% to 43% [3]. In a meta-analysis of quality of life, physical functioning was 20 points (0 to 100) and mental health 10 points below UK norms [3].

Patients are exposed to many stressors in the intensive care unit (ICU), including illness, pain, sleep deprivation, thirst, hunger, dyspnea, unnatural noise and light, inability to communicate, isolation and fear of dying; and they may show extreme emotional reactions in response [6-8]. Interventions, such as mechanical ventilation (MV) or invasive monitoring for cardiovascular support, may be difficult for patients to tolerate. Furthermore, the onset of delirium, including frightening psychotic symptoms, such as hallucinations and paranoid delusions, is common in intensive care [9,10]. Delirium is associated with the pathophysiology of critical illness as well as drugs used in intensive care [11,12]. The question is whether exposure to stressors, such as MV, or acute psychological reactions, such as stress, mood and hallucinations, are direct risk factors for PTSD and other adverse outcomes. It may be that patients' emotional reactions to stress in intensive care are early signs of psychological morbidity.

Consistent risk factors for post-ICU psychological morbidity have not been definitively established [13,14], with associations mostly detected in very few studies. Socio-demographic risk factors for post-ICU PTSD include age [15,16,18], sex [17,18] and unemployment [19]. Psychological history is a known chronic health risk factor [12,15]. Acute psychological risk symptoms (extreme fear and agitation in the ICU) were found to be associated with PTSD in only one study to our knowledge [17]. Factual recall and memory of pain were associated with PTSD in one study [19], whereas delusional memories (of psychotic symptoms) following ICU discharge were more important in others [12,20]. Clinical risk factors include aspects of sedation [12,17,18,21] and duration of mechanical ventilation [15]. Two studies that found no association with mechanical ventilation and PTSD were small, with 41 [18] and 37 [22] participants. As mechanical ventilation is the most common intensive care intervention, replication of the positive result [15] is urgently needed.

The few risk factors identified for post-ICU depression and anxiety were mainly found in single or small studies or sub-groups of patients. Hypoglycemia [23] and benzodiazepine dosage [24] were associated with post-ICU depression in patients with acute lung injury. Pessimism was associated with subsequent depression and anxiety in one study [19]. A more consistent group of risk factors (age, illness severity, ICU length of stay and prior health) were identified in studies of post-ICU quality of life [3].

The aim of our study was to investigate a broader set of clinical, acute psychological, socio-demographic and chronic health risk factors than had previously been tested, for different psychosocial outcomes within a single study of mixed general ICU patients. We used multivariable analysis to determine relative contributions of risk factors in different domains. Furthermore, we aimed to identify modifiable clinical and acute psychological risk factors that might inform the development and evaluation of preventative interventions in intensive care.

## Materials and methods

### Study design

This was a prospective cohort study with four groups of potential risk factors (clinical, acute psychological, socio-demographic and chronic health). Probable PTSD at three months was the primary outcome while depression, anxiety, and mental and physical quality of life at three months were secondary outcomes.

### Participants

The sample consisted of consecutive, highest acuity adult patients who received level three care in a large general ICU at University College Hospital, London, England between November 2008 and September 2009. In the UK, level three patients are those receiving mechanical ventilation for more than 24 hours or patients with two or more organs supported. Patients were recruited in the ICU when physicians determined they were showing signs of recovery; when they had capacity to give informed consent, and were awake, alert and able to communicate. They were not recruited on a specific day of their ICU stay, as patients woke up and became alert at different times. They were excluded if they were not English-speaking; had dementia or remained confused or had a low GCS (Glasgow Coma Scale) until their discharge from ICU; were unable to communicate until their discharge from ICU; had severe sensory impairment; or were deemed terminally ill (for example, were receiving palliative care).

### Ethics

The study was approved by the Joint University College London/University College London Hospitals Committee on the Ethics of Human Research.

### Procedure

ICU patient lists were checked daily to identify eligible participants who had received level three care during their stay. After being assessed for capacity by a health psychologist (the first author), and giving informed consent, patients completed a psychological questionnaire. Patients found to have current confusion or inability to communicate were recruited later in their stay, if and when these problems had resolved. Clinical and socio-demographic data were collected from electronic patient notes held in the ICU. Three months after discharge from the ICU, patients were sent a postal questionnaire, which included measures of PTSD, depression, anxiety, Health-Related Quality of Life (HRQL) and socio-economic circumstances.

### Data collection

Socio-demographic data recorded include age, gender, ethnicity and socio-economic position, measured using the National Statistics Socio-Economic Classification [25]. The NS-SEC is a measure of employment relations and conditions of occupations, and is the most widely used measure of socio-economic positions in official UK statistics. The self-coded version of the NS-SEC used in this study has five classes of occupation: managerial and professional; intermediate; small employers and own account workers; lower supervisory and technical; semi-routine and routine. A sixth unclassified category was added.

Clinical data include: type of admission (elective surgical, emergency surgical, non-surgical), source of admission (theatre, ward, Accident & Emergency, other), acute physiology and chronic health evaluation II score (APACHE II) [26], length of stay (days), days of organ support, type of organ support, an infection biomarker (C-reactive protein) and highest therapeutic intervention (Therapeutic Intervention Scoring System, TISS) score during the admission [27]. The TISS score reflects the type and number of intensive care interventions received, with points added for each intensive care activity. Data on drugs administered included exposure to sleep medications (mainly zopiclone), benzodiazepines, anaesthetic agents (mainly propofol), antipsychotics, inotropes and vasopressors, systemically-administered corticosteroids, and opioids; number of psychoactive drug groups received (0 to 7); and the number of days patients were sedated.

Information on "chronic health" factors (chronic physical conditions, psychological history and alcohol use) was obtained from electronic medical records held in the ICU.

### Psychological measures

All acute psychological reactions were assessed once a patient was able to respond to questions. Mood in intensive care was measured with 15 items (on anger, anxiety, depression, positive mood and confusion) from the validated Profile of Mood States [28]. Stress reactions were assessed using a newly developed 18-item intensive care stress reactions scale (ICUSS) as validated stress questionnaires did not contain items relevant to the ICU context. The ICUSS has four subscales: "physical stress" (difficulty breathing, pain, discomfort from tubes, anxiety about breathing), "delirious symptoms" (hallucinations, nightmares, disorientation, agitation), control (communication, control, confidence, information) and support (dignity, emotional support).

Memory items, (on being admitted to the ICU, the ICU stay, and presence and content of early intrusive memories in the ICU), were developed with guidance from Professor Brewin, an expert in intrusive memories and stress. The content of intrusive memories was qualitatively assessed as "factual" (real experiences in the ICU) or "unreal" (hallucinations or delusions experienced in the ICU). The validated Brief Illness Perception Questionnaire (BIPQ) [29] was used to measure patients' subjective illness perceptions including "timeline" (how long they believed their illness would last).

### Outcome measures

Three months later, PTSD symptoms were assessed using the Post-traumatic Stress Diagnostic Scale (PDS), a well-validated instrument including a 17-item severity scale [30]. We selected the PDS as it conforms to diagnostic criteria for PTSD [5] and has high diagnostic agreement with the gold-standard Structured Clinical Interview for PTSD. Using a cut-point of 18 (on a scale of 0 to 51), shown to be a highly efficient scoring method [31], the PDS severity scale has sensitivity of 0.86, specificity of 0.87 and an overall efficiency of 0.87. Participants were asked to answer questions in relation to a specific trauma (in this case, admission to intensive care) according to PDS authors' instructions. Symptoms of depression were measured with the 20-item Center for Epidemiologic Studies Depression Scale (CES-D) [32], the most widely used measure of depression in epidemiological studies, validated for intensive care patients [33] and many other populations. We used a cut-point of 19 (on a scale of 0 to 60) rather than the usual 16, as recommended to deal with the effect of somatic items in patients with medical illness [34].

We assessed anxiety at three months using a validated short form of the State-Trait Anxiety Inventory (STAI) [35], a widely used questionnaire in many populations and health conditions. We used a cut-point of 44 (range of scores 0 to 80) as recommended for studies of medically ill patients [36]. The SF-12, extensively evaluated to establish reliability and validity, was used to measure quality of life. It yields mental and physical summary scales, transformed to have a mean of 50 and SD of 10 [37]. The follow-up questionnaire included an item about current or past psychological issues but few patients answered it, so we relied on electronic medical records to obtain details of psychological history. Three months was deemed a suitable time-point to measure outcomes, including acute PTSD [5], and to examine relationships between ICU clinical and stress factors and psychological outcomes.

### Power

To obtain an initial estimate of the sample size required, a clinically significant difference in PTSD scores between two groups, defined by a binary risk factor (for example, sex), was deemed to be 10 points on the PDS [30]. For this effect size, 80% power and 5% significance, 34 patients were required in each of the two groups. As the analyses were to be carried out using multiple regression, with both continuous and categorical risk factors, the sample size needed to be inflated. With the initial sample size of 68, a correlation coefficient of 0.3 between a continuous risk factor and outcome could be detected [38]. To detect the same correlation coefficient (0.3) between a risk factor and outcome in a multiple regression model where all other variables in the model explained 30% of the total variation in outcome, calculations indicated that the sample size needed to be inflated by 40% [38]. This yielded a total sample size of 95 patients. A drop-out rate of approximately 30% was estimated on the basis of previous experience, raising the recruitment required to approximately 140 patients. During the study, the drop-out rate was higher than expected (36%) and 17 extra patients were recruited to ensure that the study retained power.

### Statistical analysis

All statistical analyses were conducted using SPSS for Windows (version 14) (SPSS Inc., Chicago, Illinois, USA).

Distributions of risk factors were assessed with frequency histograms and statistical tests for normality. Ordinary least squares regression models were used with PTSD and other outcomes treated as continuous variables. Model building was carried out in stages so that highly correlated variables (which confounded each other) were not included in the same model and to ensure parsimony of the final model. To facilitate this, four groups of risk factors (clinical, acute psychological, socio-demographic and chronic health) were pre-defined.

(i) *Univariable analysis*. In this stage of analysis, each risk factor was related to each outcome to estimate unadjusted associations. Correlations, t-tests and one-way analysis of variance were used with, respectively, continuous, binary and categorical risk factors. Spearman's rank correlation coefficients were used if continuous risk factors were not normally distributed.

(ii) *Multivariable analysis*. In recognition of the number of potential variables being tested in these analyses and the associated implications for sample size, a two-stage multivariable process was used.

*Stage one*: Separate multivariable models were built for each outcome from risk factors within each of the four groups (clinical, acute psychological, socio-demographic and chronic health) to identify the "strongest" risk factors from each group. Risk factors included in this first stage of multivariable analysis were those that showed significant unadjusted associations (*P *<0.05) with outcomes in univariable analysis. This first stage of multivariable analysis was not carried out for a group where two or fewer significant risk factors were identified in the univariable analysis. No more than eight variables were entered into a regression in this stage of multivariable analysis due to the sample size of 100 (a rule of thumb is to have 10 to 15 times more observations than variables).

*Stage two*: The strongest risk factors from each group identified in the first stage of multivariable analysis (based on an adjusted significance level of *P *<0.01), were entered in a final series of multiple regressions to assess whether factors from different groups were independent of each other (also based on a significance level of *P *<0.01). Factors were entered in the following order: socio-demographic, clinical, chronic physical, acute psychological and psychological history (at this stage of analysis, chronic factors were split up into chronic physical and psychological history). Residuals were found to be normally distributed in all multivariable models with no evidence of multicollinearity.

## Results

**Figure 1:**
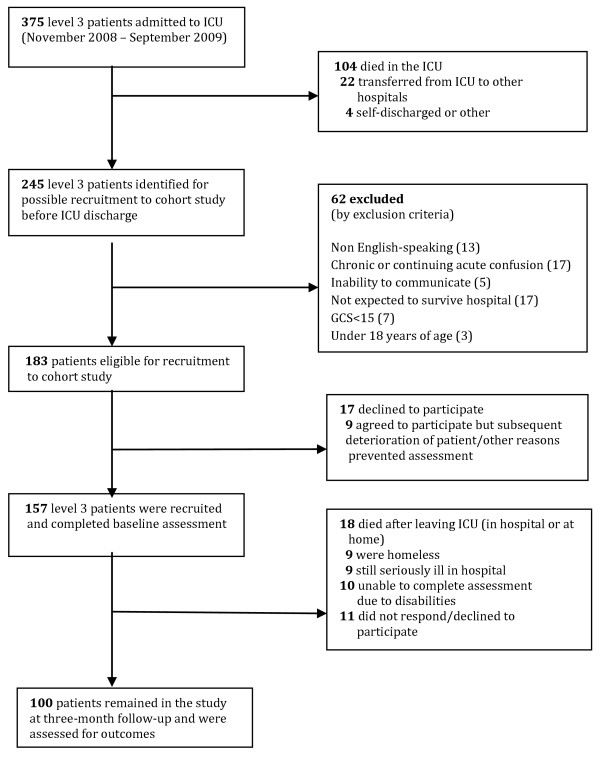
**Flow diagram of patient recruitment and participation in a cohort study of psychological outcomes of intensive care survivors**.

A total of 157 level three patients were assessed before discharge from the ICU, and 100 patients (64%) were followed up at three months (see Figure 1). Most patients were mechanically ventilated for more than 24 hours, and most were sedated with benzodiazepines or anaesthetic agents (Table 1). Patients had elevated mean scores for mood disturbance and stress reactions in ICU (Table 2). Some 65 to 75% had hallucinations, agitation and nightmares. Memory impairment, including amnesia for time spent in ICU or unwanted intrusive memories of intensive care, were common.

**Table 1:** Participants' socio-demographic and clinical characteristics

Characteristic	Followed up (n = 100)	Lost to follow-up/died (n = 57)	*P*-value
**Age - years Mean (SD)**	57.26 (17.40)	57.19 (15.62)	0.98
**Male sex, No. (%)**	52 (52%)	38 (66.7%)	0.07
**White ethnicity, No. (%)**	83 (85.6%)	49 (86%)	0.63
Occupation (by NS-SEC)*, No. (%)			
**1. Professions/managerial**	33 (33%)	No data	
**2. Intermediate professions**	10 (10%)		
**3. Self-employed**	21 (21%)		
**4. Technical/craft**	7 (7%)		
**5. Semi-routine/routine**	20 (20%)		
**6. Unclassified**	9 (9%)		
**Type admission, No. (%)**			
**Elective surgical**	23 (23%)	14 (24.6%)	0.63
**Emergency surgical**	14 (14%)	5 (8.8%)	
**Non-surgical**	63 (63 %)	38 (66.7%)	
**Apache II score † Mean (SD)**	22.01 (7.19)	22.44 (9.07)	0.76
**Hospital length of stay - days Median (range)**	27 (239)	27 (173)	0.81
**ICU length of stay - days Median (range)**	8 (85)	10 (37)	0.62
**TISS score‡, Mean (SD)**	24.61 (5.05)	24.37 (5.86)	0.79
**Number of organs supported, Mean (SD)**	4 (7)	5 (7)	<0.05
**Number (%) receiving mechanical ventilation Duration of MV in days, Median (range)**	79 (79%) 3 (80)	49 (88% ) 4 (28)	0.43
**Number (%) receiving cardiovascular support Duration of CV support in days, Median (range)**	52 (52%) 1 (16)	36 (63% ) 1 (20)	0.24
**Duration of sedation - days Median (range)**	2 (24)	2 (21)	0.18
**Benzodiazepines in ICU (yes/no), No. (%)**	60 (60%)	40 (70.2%)	0.19
**Anaesthetic agents in ICU (yes/no), No. (%)**	66 (66%)	39 (68.4%)	0.76
**Antipsychotics in ICU (yes/no), No. (%)rows from here down have less space than rows above**	39 (39%)	27 (47.4%)	0.70
**Inotropes/vasopressors in ICU (yes/no), No.(%)**	47 (47%)	35 (61.4%)	0.08
**Steroids in ICU (yes/no), No. (%)**	33 (33%)	20 (35.1%)	0.79
**Opioids in ICU (yes/no), No. (%)**	93 (93%)	53 (93%)	0.99
**Highest C-reactive protein in ICU, Mean (SD)**	212.72 (126.79)	No data	
Post-hospital destination**			<0.01
**Primary body system †† **			0.63

* NS-SEC, National Statistics Socio-economic Classification (UK) [25]

† Scores for the Acute Physiology, Age, and Chronic Health Evaluation (APACHE II) [26] range from 0 to 71; higher scores indicate more severe illness

‡ In the Therapeutic Intervention Scoring System [27] points are added for each new ICU activity

** Categories of post-hospital destination were 1. Home 2. Transfer to other hospital 3. Care or rehab centre 4. Died in hospital5. Readmission since discharge home 6. Still in hospital at three months (not yet discharged). Numbers in each category are not reported here due to lack of space

**†† **Primary body system had 11 categories: respiratory, cardiovascular, gastro-intestinal, neurological, trauma, poisoning, genito-urinary, endocrine, haematological, musculo-skeletal and dermatological. Numbers in each category are not reported here due to lack of space.

**Table 2:** Acute psychological responses in the Intensive Care Unit (ICU)

		Followed up (n = 100)	Died/lost to follow- up (n = 57)	*P*-value - difference
**(i) Total ICU mood Mean (SD) disturbance**		29.00 (13.60) Scale 0 to 60	27.18 (13.58)	*P *= 0.42
**ii) Total ICU stress Mean (SD) reactions**		32.89 (12.81) Scale 0 to 72	31.62 (11.98)	*P *= 0.54
**a) Physical stress (subscale of ICU stress)**		8.61 (4.46) Scale 0 to 16	7.57 (4.34)	*P *= 0.72
**b) Delirious symptoms (subscale of ICU stress)**		8.17 (5.04) Scale 0 to 20	7.86 (5.49)	*P *= 0.16
**iii) Illness perceptions, Mean (SD)** **a) Timeline - how long you think condition will last**		Range 0 to 10 6.64 (2.77)	6.44 (2.93)	*P *= 0.69
**b) Concern about condition**		7.34 (2.8)	7.09 (3.2)	*P *= 0.61
**c) Control over condition**		4 .00 (2.97)	4.62 (3.31)	*P *= 0.25
**d) Understanding condition**		7.06 (2.97)	7.41 (3.23)	*P *= 0.5
**e) Emotional representation of condition**		5.92 (3.4)	6.24 (3.75)	*P *= 0.59
**iv) Memory No. (%)** **a) Memory of initial admission to ICU**	Yes No	34 (34.3%) 65 (65.7%)	21(37.5%) 35 (62.5%)	*P *= 0.69
**b) Memory for whole ICU stay**	Little Some Most	45 (45.5%) 29 (29.3%) 25 (25.3%)	21 (37.5%) 13 (23.2%) 22 (39.3%)	*P *= 0.19
**c) Presence of early intrusive memories of ICU**	Yes No	49 (49.5%) 50 (50.5%)	24 (42.8%) 32 (57.1%)	*P *= 0.73
**d) Content of early intrusive memories, if experienced**	Factual Delusional Both/other*	22.6% 20% 6.9%	No data	

(i) Total mood disturbance was measured using the Profile of Mood States [28]

(ii) Total ICU stress, a) physical stress and b) delirious symptoms were measured with the ICU stress reactions scale

(iii) Illness perceptions were measured using the BIPQ [29]

*Patients had both factual and delusional memories, or did not describe the content of memories

Subsequently, the incidence of probable PTSD at three months was 27.1% (95%CI: 18.3%, 35.9%). Prevalence of probable depression was 46.3% (95% CI: 36.5%, 56.1%) and anxiety 44.4% (95% CI: 34.6%, 54.2%). In all, 55% of patients had psychological morbidity at three months. There were 16% of patients with prior history of psychological morbidity (depression in all cases). Mean mental quality of life was 43.9 (95% CI: 41.6, 46.3), six points below the population norm (50). Mean physical quality of life was 34.4 (95% CI: 32.3, 36.6), 16 points below the norm.

All psychological measures used had reliability (internal consistency), using Cronbach's alpha (0.91 for Profile of Mood States (POMS); 0.93 for PDS; O.91 for CES-D; and 0.88 for STAI). After principal components analysis, the ICU stress reactions scale was found to have four factors: physical stress, delirious symptoms, personal control and support. The total scale and three subscales were reliable (Cronbach's alphas: 0.83 (total); 0.78 (personal control); 0.74 (delirious symptoms); 0.75 (physical stress)). ICU stress reactions scores were highly correlated with POMS [28] scores (r = 0.73, *P *<0.01), suggesting concurrent validity. ICU stress reaction scores were also highly correlated with PTSD, depression and anxiety at three months, suggesting predictive validity.

### PTSD

Because of the number of risk factors and outcomes investigated, the full three-stage statistical analysis is reported for PTSD only in the main paper. However, the same process was used for each outcome (see Tables 3 and 4 for univariable analyses of secondary outcomes, and Additional file 1 for further multivariable analyses tables).

**Table 3:** Unadjusted associations between clinical factors and psycho-social outcomes three months after intensive care

	Post-traumatic stress disorder*	Depression	Anxiety	Mental quality of life	Physical quality of life
**TISS (Therapeutic intervention scoring) **	0.25 . *P *= 0.01	0.08 *P *= 0.44	0.07 *P *= 0.52	-0.06 *P *= 0.62	0.04 *P *= 0.74
**Number of organs supported**	0.26 *P *<0.01	0.12 *P *= 0.23	0.06 *P *= 0.57	-0.08 *P *= 0.47	0.08 *P *= 0.49
**Duration of sedation**	0.27 *P *<0.01	0.19 *P *= 0.07	0.17 *P *= 0.09	-0.20 *P *= 0.06	0.03 *P *= 0.82
**Number of drug groups**	0.28 *P *<0.01	0.10 *P *= 0.32	0.10 *P *= 0.31	-0.10 *P *= 0.47	-0.20 *P *= 0.07
**Length of stay in ICU**	0.11 *P *= 0.29	-0.05 *P *= 0.66	-0.06 *P *= 0.58	-0.02 *P *= 0.87	0.02 *P *= 0.87
**Length of hospital stay**	0.15 *P *= 0.15	0.21 *P *<0.05	0.09 *P *= 0.39	-0.18 *P *= 0.12	-0.07 *P *= 0.56
**Type of admission†**	*P *= 0.81	*P *= 0.50	*P *= 0.23	*P *= 0.81	*P *= 0.53
**Post-hospital destination**	*P *= 0.38	*P *<0.05	*P *= 0.25	*P *= 0.22	*P *= 0.90
**Primary body system**	*P *= 0.20	*P *= 0.03	*P *= 0.14	*P *= 0.30	*P *= 0.17
**Duration of mechanical ventilation**	0.20 *P *<0.05	0.09 *P *= 0.39	0.06 *P *= 0.57	-0.013 *P *= 0.91	-0.014 *P *= 0.90
**Duration of cardiovascular support**	0.25 *P *<0.05	0.14 *P *= 0.17	0.13 *P *= 0.22	-0.20 *P *= 0.06	-0.03 *P *= 0.79
**Benzodiazepines ‡**	6.96 (2.36, 11.57) *P *<0.01	7.44 (1.81, 13.07) *P *= 0.01	5.95 (0.03, 11.87) *P *<0.05	-4.08 (-8.73, .56) *P *= 0.08	-.27 (-4.67, 4.12) *P *= 0.90
**Anaesthestics**	1.64 (-3.35, 6.65) *P *= 0.51	-2.35 (-8.50, 3.80) *P *= 0.45	-2.61 (-8.88, 3.65) *P *= 0.41	2.02 (-2.9, 6.93) *P *= 0.42	4.45 (-0.04, 8.94) *P *= 0.05
**ll Inotropes or vasopressors**	4.84 (0.1, 9.57) *P *<0.05	3.70 (1.99, 9.40) *P *= 0.20	7.63 (0.89, 13.37) *P *= 0.01	-4.51 (-9.08, .06) *P *= 0.05	0.06 (-4.29, 4.41) *P *= 0.98
**Antipsychotics**	5.81 (0.8, 10.81) P <0.05	1.59 (-4.31, 7.39) *P *= 0.59	1.18 (-4.87, 7.25) *P *= 0.70	-1.58 (-6.28, 3.12) *P *= 0.51	4.14 (-0.15, 8.43) P = 0.06
**Opioids**	-0.55 (-10.42, 9.32) *P *= 0.91	-7.12 (-18, 3.77) *P *= 0.20	-7.79 (-19.25, 3.66) *P *= 0.18	7.42 (-0.96, 15.8) *P *= 0.08	0.29 (-7.65, 8.23) *P *= 0.94
**Steroids**	-0.28 (-5.33, 4.77) *P *= 0.91	-1.08 (-7.25, 5.08) *P *= 0.73	-1.57 (-7.85, 4.71) *P *= 0.62	-.59 (-5.48, 4.31) *P *= 0.81	5.57 (1.18, 9.96) *P *<0.05
**Highest C-reactive protein in ICU**	0.22 *P *<0.05	0.10 *P *= 0.32	0.08 *P *= 0.44	No data	No data

Effect sizes presented in the table are Pearson's r for normally distributed exposure variables, Spearman's rho for skewed variables, and mean difference with 95% CI for binary variables.

* Range of scores for outcome measures: PTSD 0-51 (PDS); depression 0 to 60 (CES-D); anxiety 0 to 80 (STAI); mental quality of life 0 to 100 (SF-12 mental component summary score); physical quality of life 0 to 100 (SF-12 physical component summary score)

† For type of admission, post-hospital destination and primary body system (categorical variables ) the *P*-values of the F statistic are presented.

‡ For all drug variables, the mean score of patients who did not receive the drug group was subtracted from the mean score of patients who did receive the drug.

**Table 4:** Unadjusted associations between socio-demographic variables, acute psychological reactions in ICU, and three month outcomes

	PTSD*	Depression	Anxiety	Mental quality of life	Physical quality of life
**Age**	-0.18 *P *= 0.07	0.17 *P *= 0.11	-0.03 *P *= 0.79	0.10 *P *= 0.38	-0.10 *P *= 0.38
**Sex (female - male)**	4.30 (-0.44, 9.04) *P *= 0.08	4.01 (1.68, 9.69) *P *= 0.17	-3.39 *P *= 0.26	4.20 (-0.39, 8.79) *P *= 0.07	-0.81 (-5.16, 3.54) *P *= 0.71
**Ethnicity (white/other)**	*P *= 0.60	*P *= 0.05	*P *= 0.49	*P *= 0.20	*P *= 0.15
**Socio-economic position** 1. Professions/managerial 2. Intermediate profession 3. Self-employed 4. Technical/craft 5. Semi-routine/routine 6. Unclassified	*P *= 0.25 11.77 (9.10) 15.61 (7.25) 13.12 (12.46) 8.04 (4.79) 18.19 (13.77) 18.56(18.66)	*P *<0.01 14.46 (10.68) 30.33 (12.51) 22.71 (14.21) 13.64 (8.59) 24.75 (15.65) 24.98 (21.30)	*P *<0.05 39.27 (11.69) 53.00 (13.19) 43.33 (13.89) 36.19 (11.45) 46.33 (16.11) 50.52 (21.30)	*P *<0.05 48.47 (9.53) 39.58 (10.73) 45.55 (10.79) 43.53 (8.75) 38.43 (9.48) 37.98 (12.97)	*P *= 0.69 35.86 (9.66) 31.16 (6.95) 34.13 (10.81) 38.21 (10.77) 35.40 (11.32) 34.57 (11.45)
**ICU mood † disturbance**	0.50 *P *<0.01	0.42 *P *<0.01	0.38 P <0.01	-0.47 *P *<0.01	-0.01 *P *= 0.92
**ICU stress reactions**	0.60 *P *<0.01	0.36 *P *<0.01	0.32 *P *<0.01	-0.37 *P *<0.01	-0.90 *P *= 0.41
**ICU delirious symptoms**	0.40 *P *<0.01	0.25 *P *= 0.01	0.20 *P *= 0.05	-0.27 *P *= 0.01	0.00 *P *= 0.99
**ICU memory‡ (little memory - some/most memory)**	6.30 (1.56, 10.98) *P *= 0.01	6.05 (0.37, 11.73) *P *<0.05	3.06 (-2.91, 9.03) *P *= 0.31	-2.01 (-6.71, 2.68) *P *= 0.40	0.54 (-3.85, 4.95,) *P *= 0.81
**ICU Intrusive memories (some - none)**	9.39 (4.92, 13.85) *P *<0.01	7.10 (1.47, 12.71) *P *= 0.01	5.85 (-0.02, 11.02) *P *= 0.05	-3.38 (-8.03, 1.27) *P *= 0.15	1.86 (-2.52, 6.23) *P *= 0.40
**Illness perceptions Timeline§**	0.28, *P *<0.01	0.22, *P *= 0.04	0.23, *P *= 0.03	-0.16, *P *= 0.16	-0.39, *P *<0.01
**Concern**	0.28 *P *<0.01	0.32 *P *<0.01	0.22 *P *<0.05	-0.2 *P *= 0.07	-0.26 *P *<0.05
**Emotional representation**	0.29 *P *<0.01	0.31 *P *<0.01	0.29 *P *<0.01	-0.28 *P *<0.01	-0.18 *P *= 0.11

Could these be fitted under the table like other tables? Effect sizes presented in the table are Pearson's 'r' or Spearman's 'rho' for continuous exposure variables, or mean differences + 95% CI for binary variables.

Range of scores (outcomes): PTSD 0 to 51 (PDS); depression 0 to 60 (CES-D); anxiety 0 to 80 (STAI); mental quality of life 0 to 100 (SF-12 mental component summary score); physical quality of life 0 to 100 (SF-12 physical component summary score)

*There were significant differences in depression scores between National Statistics Socio-economic Classification (NS-SEC) classes 1 and 2. There were no significant differences in anxiety between classes, although there was an overall effect of class. There was a significant difference of mean mental HRQL between NS-SEC classes 1 and 5.

† Range of scores (risk factors): mood 0 to 60 (POMS); stress 0 to 72 (ICUSS); delirious symptoms 0 to 20 (ICUSS).

‡ ICU memory was used as a binary variable (little memory or some/most memory of ICU). Patients who had little memory of ICU had higher PTSD, depression and anxiety scores (more psychological morbidity) than patients with some/most memory of ICU. ICU intrusive memories (had or did not have intrusive memories) was also a binary variable. Patients with intrusive memories had higher PTSD, depression and anxiety scores (more psychological morbidity) than patients with intrusive memories.

§ BIPQ, Brief illness perception questionnaire. BIPQ Timeline represents how long a person believes their condition will last.

### Univariable analysis - PTSD

Clinical risk factors significantly associated with PTSD were higher TISS scores, number of organs supported, days of mechanical ventilation, days of advanced cardiovascular support, days of sedation, number of drug groups and C-reactive protein during admission; and use of benzodiazepines, inotropes/vasopressors and anti-psychotics (see Table 3). Significant acute psychological risk factors for PTSD were total mood disturbance in ICU, ICU stress reactions (including delirious symptoms), loss of memory in ICU, early intrusive memories in ICU and three illness perceptions (Table 4). Patients with ICU memory loss were more likely to have early intrusive memories (62% vs 39%, *P *<0.05). No socio-demographic factors were significantly associated with PTSD. Psychological history and alcohol use were significant "chronic health" risk factors.

### Multivariable analysis (stage one) - PTSD

All significant factors identified by univariable analysis were now entered into three separate regressions, according to group (there was no socio-demographic group for PTSD). Within the clinical group, the strongest risk factors for PTSD were days of sedation, use of benzodiazepines, use of antipsychotics and use of inotropes or vasopressors (see Additional file 1, Table S1). Within the acute psychological group, the strongest risk factors were total ICU mood, intrusive memories and perceived illness timeline (Additional file 1, Table S2). Within the chronic health group, psychological history and alcohol use remained significant (Additional file 1, Table S3).

### Multivariable analysis (stage two) - PTSD

The strongest risk factors from the groups identified by stage one multivariable analysis were now entered together in a final multiple regression (Table 5). As there were nine variables, the weakest of the four clinical factors (inotropes) was not included in this regression. Increasing duration of sedation was shown to be the strongest clinical risk factor for PTSD (Table 5, column 1). Overall, the strongest independent risk factors for PTSD were three acute psychological factors (ICU mood, intrusive memories and perceived illness timeline) and a chronic factor, psychological history (Table 5, column 3).

**Table 5:** Final multiple regression models of strongest* risk factors for post-ICU PTSD at three months

	R^2^ Cumulative variance	Clinical factors† (column 1)		Clinical and acute psychological factors (column 2)		Clinical, acute psychological and chronic psychological factors (column 3)	
		Unstandardised coefficients (95% CI)	*p*	Unstandardised coefficients (95% CI)	*p*	Unstandardised coefficients (95% CI)	*p*
**Duration of sedation - days**		0.69 (0.12, 1.27)	<0.05	0.35 (-0.17, 0.87)	0.18	0.33 (-0.18, 0.84)	0.20
**Benzodiazepines** **(yes/no)**		3.98 (-1.01, 8.97)	0.12	1.26 (-3.22, 5.73)	0.58	0.352 (-4.01,4.72)	0.87
**Antipsychotics** **(yes/no)**		3.32 (-1.61, 8.24)	0.18	1.88 (-2.47, 6.24)	0.39	1.06 (-3.18, 5.29)	0.62
	0.18 (18%)						
**ICU Mood**				0.31 (0.14, 0.47)	<0.01	0.25 (0.09, 0.42)	<0.01
**BIPQ (timeline)**				0.79 (0.05, 1.52)	<0.05	0.71 (-0.003, 1.43)	0.05
**Intrusions** **(yes/no)**				5.21 (0.91, 9.51)	<0.05	5.83 (1.65, 10.02)	<0.01
	0.39 (39%)						
**Psychological history** **(yes/no)**						6.55 (0.99, 12.10)	<0.05
**Alcohol use (yes/no)**						4.63 (-1.51, 10.77)	0.14
	0.45 (45%)						

PTSD scores range from 0 to 51 on the post-traumatic stress diagnostic scale (PDS).

* Strongest risk factors were identified in a previous univariable analysis and separate multivariable analyses of each group of risk factors (clinical, acute psychological, chronic health)

† Factors were entered in this final multiple regression in the following order: 1. Clinical, 2. acute psychological, 3. chronic psychological. There are no socio-demographic factors or chronic physical conditions in this table as neither S-D factors nor chronic physical conditions had significant associations with PTSD in the univariable analysis.

BIPQ, Brief Illness Perception Questionnaire

### Depression and anxiety

For secondary outcomes, depression and anxiety, only stage two of multivariable analysis is reported here. See Additional file 1, Tables S4-S6 for stage one multivariable analyses carried out for depression and anxiety.

### Stage two multivariable analysis - depression

Receiving benzodiazepines in intensive care was the strongest clinical risk factor for depression (Table 6, column 1) after adjusting for socio-demographic factors. ICU mood, socio-economic position and psychological history were the strongest independent risk factors for depression in the fully adjusted model (Table 6, column 3).

**Table 6:** Final multiple regression models of strongest* risk factors for post-ICU depression at three months

	R^2^ Cumulative variance	Socio-demographic (S-D), clinical and chronic physical factors† (column 1)		S-D, clinical, chronic physical and acute psychological factors (column 2)		S-D, clinical, chronic physical, acute psychological and chronic psychological (column 3)	
		Unstandardised coefficients (95% CI)	*P*	Unstandardised coefficients (95% CI)	*P*	Unstandardised coefficients (95% CI)	*P*
**Ethnicity (white/other)**		5.34 (-1.40, 12.07)	0.12	3.78 (-2.66, 10.21)	0.25	5.15 (-1.17, 11.47)	0.11
**SEC2‡**		14.59 (4.71, 24.46)	<0.01	10.42 (0.76, 20.08)	<0.05	11.39 (2.04, 20.75)	<0.05
**SEC3**		7.86 (0.61, 15.12)	<0.05	8.40 (1.53, 15.26)	<0.05	7.61 (0.95, 14.27)	<0.05
**SEC4**		-1.75 (-12.14, 8.64)	0.74	-1.68 (-11.51, 8.14)	0.74	-0.38	0.94
**SEC5**		9.08 (1.93, 16.23)	<0.05	10.74 (3.90, 17.57)	<0.01	10.55 (3.95, 17.14)	<0.01
**SEC6**		7.64 (-2.08, 17.36)	0.12	7.67 (-1.52, 16.86)	0.10	7.40 (-1.46 16.26)	0.11
**Benzodiazepines** **(yes/no)**		6.73 (1.42, 12.05)	<0.05	4.54 (-0.65, 9.73)	0.09	3.80 (-1.24, 8.85)	0.14
**Chronic physical health (yes/no)**		5.05 (-0.20, 10.31)	0.06	2.82 (-2.32, 7.96)	0.28	3.10 (-1.86, 8.06)	0.22
	0.27 (27%)						
**ICU Mood**				0.35 (0.14, 0.55)	<0.01	0.28 (0.07, 0.48)	<0.01
	0.36 (36%)						
**Psychological history (yes/no)**						7.67 (0.86, 14.48)	<0.05
	0.39 (39%)						

Depression was measured using the Center for Epidemiologic Studies Depression Scale (CES-D, range of scores 0 to 60).

* Strongest risk factors were identified in a previous univariable analysis, followed by multivariable analyses of each group of risk factors (socio-demographic, clinical, acute psychological, chronic health).

† Factors were entered in this final multiple regression in the following blocks: 1. Socio-demographic 2. Clinical3. Chronic physical conditions 4. Acute psychological; 5. Chronic psychological (each stage is not shown in a separate column for space reasons).

‡ Variables SEC2-SEC6 are dummy variables representing differences between occupational categories within the National Statistics Socio-economic classification (NS-SEC). In each dummy variable the numbered category is compared with the baseline of category 1. (NS-SEC categories are as follows: 1. Professions/managerial2. Intermediate professions3. Self-employed 4. Technical/craft 5. Semi-routine/routine 6. Unclassified)

### Stage two multivariable analyses - anxiety

Receiving inotropes or vasopressors was the strongest clinical risk factor for higher anxiety (Table 7, column 1). Socio-economic position, chronic physical health, ICU mood and psychological history were the strongest independent risk factors for anxiety in the final model (Table 7, column 3).

**Table 7:** Final multiple regression models of strongest* risk factors for post-ICU anxiety at three months

	R^2^ Cumulative variance explained	Socio-demographic, clinical and chronic physical factors† (column 1)		Socio-demographic, clinical, chronic physical and acute psychological factors (column 2)		Socio-demographic, clinical, chronic health, acute psychological and psychological history (column 3)	
		Unstandardised coefficients (95% CI)	*P*	Unstandardised coefficients (95% CI)	*P*	Unstandardised coefficients (95% CI)	*P*
							
**SEC2‡**		11.52 (1.50, 21.55)	<0.05	7.82 (-2.47, 18.10)	0.13	9.35 (-0.81, 19.52)	0.07
**SEC3**		5.87 (-2.06, 13.80)	0.15	5.92 (-1.84, 13.68)	0.13	4.73 (-2.94, 12.41)	0.22
**SEC4**		-2.17 (-13.37, 9.03)	0.70	-2.45 (-13.41, 8.51)	0.66	-1.37 (-12.15, 9.40)	0.80
**SEC5**		7.81 (-0.08, 15.7)	<0.05	8.14 (0.223, 16.05)	<0.05	7.99 (0.24, 15.73)	<0.05
**SEC6**		10.66 (0.37, 20.94)	<0.05	9.57 (-0.69, 19.82)	0.07	9.76 (-0.29, 19.80)	0.06
**Inotropes or vasopressors (yes/no)**		6.60 (0.67, 12.53)	<0.05	5.48 (-0.46, 11.42)	0.07	4.64 (-1.23, 10.50)	0.12
**Benzodiazepines** (yes/no)		4.06 (-1.87, 9.99)	0.18	2.59 (-3.34, 8.52)	0.39	2.08 (-3.74, 7.90)	0.48
**Chronic physical health (yes/no)**		6.57 (0.95, 12.12)	<0.05	4.62 (-1.14, 10.37)	0 .1	5.16 (-0.50, 10.81)	0.07
	0.25 (25%)						
**Mood**				0.26 (0.04, 0.49)	<0.05	0.20 (-0.03, 0.428)	0.09
**Timeline (BIPQ)**				0.36 (-0.70, 1.43)	0.50	0.18 (-0.87, 1.24)	0.73
	0.30 (30%)						
							
**Psychological history (yes/no)**						8.37 (0.67, 16.08)	<0.05
	0.34 (34%)						

Anxiety was measured using the State-trait anxiety inventory (STAI), range of scores 0 to 80

* Strongest risk factors were identified in a previous univariable analysis, followed by multivariable analysis of each group of risk factors (socio-demographic, clinical, acute psychological, chronic health).

† Factors were entered in this final multiple regression in the following order: 1. Socio-demographic 2. Clinical 3. Chronic physical conditions 4. Acute psychological 5. Psychological history (each stage is not shown in a separate column for space reasons)

‡ Variables SEC2-SEC6 are dummy variables representing differences between occupational categories within the National Statistics Socio-economic classification (NS-SEC). In each dummy variable the numbered category is compared with the baseline of category 1. (NS-SEC categories are: 1. Professions/managerial 2. Intermediate professions 3. Self-employed 4. Technical/craft 5. Semi-routine/routine 6. Unclassified

### Multivariable analyses - quality of life (mental component)

As relatively few risk factors were identified for quality of life in univariable analyses, only one stage of multivariable analysis was necessary (Additional file 1, Table S7). Use of inotropes or vasopressors was the strongest clinical risk factor for worse mental quality of life (mean difference = -4.21 points on the SF-12 mental summary scale (95% CI: -8.45, 0.03)). ICU mood, chronic physical health and socio-economic position were the strongest independent risk factors for mental quality of life in the fully adjusted model.

### Multivariable analysis - quality of life (physical component)

Better physical quality of life was most strongly associated with ICU steroid usage (mean difference = 4.81 points on the SF-12 physical summary scale, (95% CI: 1.66, 9.27) *P *<0.05). Steroids confounded the effect of chronic physical conditions on physical quality of life. Use of anaesthetic agents and the illness perception "timeline" were also independent predictors of better physical quality of life (see Additional file 1, Table S8).

### Relative contributions of risk factors

In the final regression models for PTSD, depression, anxiety (see Tables 5, 6, 7) and mental quality of life (Additional file 1, Table S7), the strongest clinical risk factors became weaker (effect sizes or unstandardised coefficients were reduced by up to a half) and were non-significant when acute psychological factors were added. This suggests that acute psychological reactions partially explained (or mediated) the effects of clinical risk factors on psychological outcomes. Additional mediational analyses carried out, but not reported here, confirmed that most associations between clinical risk factors and outcomes were mediated by acute psychological risk factors. Background factors, such as socio-economic position and chronic health (physical and psychological), were also strong, independent risk factors of psycho-social outcomes but did not confound the effects of acute psychological reactions in intensive care.

## Discussion

In this prospective study, we found that level three patients with mixed diagnoses suffer considerable psychological distress both during and following a general ICU admission. Three months after being discharged, 27% had probable PTSD symptoms, 46% had probable depression and 44% had anxiety. Our PTSD estimate is broadly consistent with a systematic review in which median point prevalence of PTSD was 22% [1] and the expectation that 25 to 30% of people develop PTSD after a trauma [39]. Post-ICU depression and anxiety rates were high in this study, compared to 28% depression reported in a systematic review [2] and anxiety rates varying from 5 to 43% [3]. The varying rates of morbidity may be explained by differences in populations, admission criteria, and methods and timing of assessments. We believe our prevalence estimates are credible due to the high quality of questionnaires used to measure psychological morbidity and the representativeness of our level three samples.

Patients had high mean scores for mood disturbance and stress (see Table 2) in response to sleep deprivation, difficulty breathing, pain, inability to communicate, low control, hallucinations and nightmares. These stress reactions were measured during their ICU admission. Previous studies measured stress in ICU retrospectively [17] or in sub-groups, such as chronically critically ill [6] or terminally ill [8] patients. The presence of delirium has been well documented in intensive care patients [9,40]. In this study, we were interested in measuring specific delirium symptoms, such as hallucinations, nightmares and agitation, which we found to be at high levels.

Acute psychological risk factors for PTSD, identified in univariable analysis, include higher intensive care stress and delirious symptom scores (measured using the ICUSS). Associations were also found between ICU stress and delirious symptoms, and subsequent depression. However, in spite of moderate to large effect sizes, ICU stress and delirious symptoms were confounded by the variable ICU mood in the first stage of multivariable analysis. ICU mood and stress may have been overlapping variables [41] with mood showing slightly larger effect sizes. As one sub-scale in the ICU stress reactions scale was found unreliable and did not correlate with outcomes, omitting this sub-scale might increase the scale's utility in future.

The strongest acute psychological risk factors for PTSD identified in multivariable analysis were mood in the ICU, the perceived timeline of illness and early intrusive memories of intensive care. The strongest acute psychological risk factor for depression was also mood in the ICU. This mood variable was composed of symptoms, such as anger, nervousness, low mood and confusion. The first three are common stress reactions while the latter is arguably related to hypoxia, sedation or delirium. The identification of ICU mood as one of the strongest risk factors in the study suggests that emotional stress reactions in intensive care may be a trigger for, or early manifestation of, future psychological morbidity.

It was of interest that early intrusive memories in intensive care were associated with memory loss. Patients who remembered little of their ICU stay were more likely to have early intrusive memories than those who remembered more. It is known that periods of unconsciousness do not preclude the development of intrusive memories [42]. Other ICU studies emphasise the relationship between "delusional" memories and PTSD [12,20] but in our study there was no significant difference in outcomes between patients with factual or delusional intrusive memories. There is no consensus in the wider PTSD literature about the significance of early intrusive memories that immediately follow a trauma. Some studies predict successful recovery, but others predict a worse outcome [39].

Turning to clinical risk factors, it was of interest that many variables, such as TISS score [27], duration of mechanical ventilation and cardiovascular support, number of organs supported, drug groups given and length of sedation, were associated with PTSD in the univariable analysis. These results suggest that a level three admission, particularly when it involves multiple drugs and escalating invasive interventions, may be a traumatic stressor that can trigger PTSD symptoms if the patient survives.

During the first stage of multivariable analysis it emerged that the strongest clinical risk factors for PTSD were drug-related variables, particularly the number of days of sedation. In previous studies, PTSD was found to be associated with other aspects of sedation [12,17,18,21]. Our study also found strong associations in the first stage of multivariable analysis between other ICU drugs and psychological outcomes, including benzodiazepines and depression; inotropes/vasopressors and anxiety; and both steroids and anaesthetic agents (mainly propofol) with improved physical quality of life.

It has been hypothesised that benzodiazepines trigger depression by reducing central monoamine activity [43]. The association between benzodiazepine use in the ICU and delirium [11,12] also suggests pathways leading to long-term psychological morbidity. The association between inotropes and vasopressors in intensive care and subsequent anxiety has not previously been reported, although receiving noradrenaline or adrenaline was associated with short-term anxiety in medical patients [44]. These medications are known to enhance emotional memories [39], which are prominent in anxiety disorders. However, patients receiving inotropes and vasopressors are at risk for inadequate brain perfusion. Therefore, it should not be assumed the association is causal.

Regarding the association between corticosteroids and improved physical quality of life, it could be hypothesised that steroids offer protection by modifying the inflammatory response. In another study, patients receiving steroids in intensive care had a lower rate of PTSD [45]. However, caution is needed as the use of corticosteroids in intensive care has previously been associated with long-term physical impairments [46].

Few socio-demographic risk factors were identified in the analyses, perhaps suggesting that the stressful effects of intensive care transcend age or gender. However, lower socio-economic position was found to predict depression, anxiety and mental quality of life, although not PTSD. It may be that PTSD symptoms are directly triggered by traumatic experiences in intensive care, while depression and anxiety at three months are more affected by socio-economic factors. No previous studies of psychological outcomes after intensive care included a valid measure of socio-economic position, although this has been shown to predict mortality in ICU patients [4,47].

The most important finding in this study was that acute psychological reactions were among the strongest risk factors for post-ICU psychological morbidity. The second stage of multivariable analysis demonstrated that associations between clinical factors, such as duration of sedation, and outcomes, such as PTSD, were weakened when acute psychological factors were added to the regression. This suggests that the effects of clinical factors on outcomes were partially explained (or mediated) by acute psychological reactions. It is important to note that the effects of acute stress reactions in the ICU on outcomes were not confounded by psychological history. Thus, stress in the ICU was found to contribute to future psychological morbidity independently of pre-existing psychological problems.

These results suggest that, as well as modifying clinical and sedation practices in the ICU, psychological interventions aiming to mitigate acute stress reactions in intensive care might have a positive impact on poor psycho-social outcomes.

The strengths of this study include the measurement of several important psychological outcomes with validated questionnaires, and of a comprehensive set of risk factors. The prospective design and participation of a representative sample of highest acuity "level three" general ICU patients, who are difficult to recruit, are also positive aspects of the study. The study was fully powered to detect associations between risk factors and outcomes using multiple regression models.

Limitations include the use of a single-centre. Another limitation was the necessary exclusion of patients who remained confused throughout the intensive care admission. Psychological questionnaires were used rather than clinician diagnosis of outcome. The ICU Stress Reactions Scale (ICUSS) was not validated before the study. However, this innovative instrument enables the measurement of ICU stress reactions in real time, not retrospectively, and preliminary validational data for the scale were collected. Records of patient's past medical history may not have been complete. The loss of 36% of participants to follow-up was due to death, homelessness, disability and hospitalisation. However, 90% of the patients who were able to participate in follow-up, completed the study.

## Conclusions

This cohort study revealed that level three patients suffered considerable psychological morbidity after intensive care. We detected associations not found in previous studies: between inotropes/vasopressors and post-ICU anxiety; corticosteroids and better physical quality of life; and between delirious symptoms, early intrusive memories and memory loss with depression and PTSD. Our results lend weight to limited existing evidence that sedation is linked to depression and PTSD after intensive care. It was striking that different drug-related clinical risk factors were correlated with different outcomes, and further studies to assess mechanisms are warranted. The most important finding was that acute stress reactions in the ICU were stronger risk factors than clinical factors. This lends hope that modifying psychological as well as pharmacological risk factors may be possible, and preventative approaches to ICU stress could be developed and evaluated.

## Key messages

• High rates of psychological morbidity were found among level three patients three months after intensive care: PTSD (27%), depression (46%) and anxiety (44%).

• Strong acute psychological reactions in intensive care were among the risk factors most strongly associated with later psychological morbidity.

• Clinical risk factors for poor psychosocial outcomes included duration of sedation (PTSD); use of benzodiazepines (depression); inotropes and vasopressors (anxiety) and corticosteroids (better physical quality of life).

• The correlation of different clinical risk factors with different psychosocial outcomes suggests that investigations of psychobiological mechanisms are warranted.

• The risk factors identified suggest that psychological interventions, as well as pharmacological modifications, have the potential to reduce poor outcomes after intensive care.

## Abbreviations

APACHE: Acute Physiology and Chronic Health Evaluation; BIPQ: Brief Illness Perception Questionnaire; CES-D: Center for Epidemiologic Studies Depression Scale; DSM-IV: Diagnostic and Statistical Manual of Mental Disorders (4^th ^Ed); GCS: Glasgow Coma Scale; HRQL: Health-Related Quality of Life; ICU: Intensive care unit; ICUSS: Intensive Care Stress Reactions Scale; MV: Mechanical ventilation; NS-SEC: National Statistics Socio-Economic Classification; PDS: Post-traumatic Stress Diagnostic Scale; PTSD: Post-traumatic stress disorder; POMS: Profile of Mood States; STAI: State-Trait Anxiety Inventory; TISS: Therapeutic Intervention Scoring System

## Competing interests

The authors declare that they have no competing interests.

## Authors' contributions

DW conducted the study under the academic supervision of RR, JW, RH and CB. RH led the design of the analysis plan. DH and MM contributed to study design, and provided clinical support and advice at every stage of the project. SB and CM advised on the inclusion of ICU clinical risk factors and helped DW with data collection. DW drafted the initial report and the other authors revised it. All authors read and approved the final version of the paper.

## Supplementary Material

Additional file 1 **Tables showing full multivariable analyses**. Tables showing the first stage of multivariable analyses for PTSD, depression and anxiety, and full multivariable analyses for physical and mental quality of life outcomes. Click here for file

## References

[B1] Davydow DS Gifford JM Desai SV Needham DM Bienvenu OJ Posttraumatic stress disorder in general intensive care unit survivors: a systematic review Gen Hosp Psychiat 2008 16 421 443 10.1016/j.genhosppsych.2008.05.006 PMC2572638 18774425

[B2] Davydow DS Gifford JM Desai SV Bienvenu OJ Needham DM Depression in general intensive care unit survivors: a systematic review Intens Care Med 2009 16 796 809 10.1007/s00134-009-1396-5 PMC10885708 19165464

[B3] Wade D Raine R Weinman J Hardy R Tupprasoot R Mythen M Howell DC What determines poor psychological outcomes after admission to the intensive care unit? The Intensive Care Society State of the Art meeting. conference proceedings, December 2010 2010 London: The Intensive Care Society

[B4] Hutchings A Raine R Brady A Wildman M Rowan K Socioeconomic status and outcome from intensive care in England and Wales: a prospective cohort study Med Care 2004 16 943 951 10.1097/00005650-200410000-00002 15377926

[B5] American Psychiatric Association Diagnostic and Statistical Manual of Mental Disorders 1994 4 Washington DC: American Psychiatric Association

[B6] Nelson JE The symptom burden of chronic critical illness Crit Care Med 2004 16 1527 1534 10.1097/01.CCM.0000129485.08835.5A 15241097

[B7] Novaes MA Aronovich A Ferraz MB Knobel E Stressors in ICU: patients' evaluation Intens Care Med 1997 16 1282 1285 10.1007/s001340050500 9470087

[B8] Puntillo KA Arai S Cohen NH Gropper MA Neuhaus J Paul SM Miaskowski C Symptoms experienced by intensive care unit patients at high risk of dying Crit Care Med 2010 16 2155 2160 10.1097/CCM.0b013e3181f267ee 20711069 PMC3377582

[B9] Ely EW Siegel MD Inouye SK Delirium in the intensive care unit: an under-recognized syndrome of organ dysfunction Semin Respir Crit Care Med 2001 16 115 126 10.1055/s-2001-13826 16088667

[B10] Granberg A Engberg IB Lundberg D Acute confusion and unreal experiences in intensive care patients in relation to the ICU syndrome. Part II Intens Crit Care Nurs 1999 16 19 33 10.1016/S0964-3397(99)80062-7 10401338

[B11] Pandharipande P Shintani A Peterson J Pun BT Wilkinson GR Dittus RS Bernard GR Ely EW Lorazepam is an independent risk factor for transitioning to delirium in intensive care unit patients Anesthesiology 2006 16 21 26 10.1097/00000542-200601000-00005 16394685

[B12] Jones C Backman C Capuzzo M Flaatten H Rylander C Griffiths RD Precipitants of post-traumatic stress disorder following intensive care: a hypothesis generating study of diversity in care Intens Care Med 2007 16 978 985 10.1007/s00134-007-0600-8 17384929

[B13] Griffiths J Fortune G Barber V Duncan Young J The prevalence of post traumatic stress disorder in survivors of ICU treatment: a systematic review Intens Care Med 2007 16 1506 1518 10.1007/s00134-007-0730-z 17558490

[B14] Hopkins RO Key CW Suchyta MR Weaver LK Orme JF Jr Risk factors for depression and anxiety in survivors of acute respiratory distress syndrome Gen Hosp Psychiat 2010 16 147 155 10.1016/j.genhosppsych.2009.11.003 20302988

[B15] Cuthbertson BH Hull A Strachan M Scott J Post-traumatic stress disorder after critical illness requiring general intensive care Intens Care Med 2004 16 450 455 10.1007/s00134-003-2004-8 12961065

[B16] Scragg P Jones A Fauvel N Psychological problems following ICU treatment Anaesthesia 2001 16 9 14 10.1046/j.1365-2044.2001.01714.x 11167429

[B17] Samuelson KA Stressful memories and psychological distress in adult mechanically ventilated intensive care patients - a 2-month follow-up study Acta Anaesth Scand 2007 16 671 678 10.1111/j.1399-6576.2007.01292.x 17567267

[B18] Girard TD Shintani AK Jackson JC Gordon SM Pun BT Henderson MS Dittus RS Bernard GR Ely EW Risk factors for post-traumatic stress disorder symptoms following critical illness requiring mechanical ventilation: a prospective cohort study Crit Care 2007 16 R28 10.1186/cc5708 17316452 PMC2151865

[B19] Myhren H Ekeberg O Toien K Karlsson S Stokland O Posttraumatic stress, anxiety and depression symptoms in patients during the first year post intensive care unit discharge Crit Care 2010 16 R14 10.1186/cc8870 20144193 PMC2875529

[B20] Jones C Griffiths RD Humphris G Skirrow PM Memory, delusions, and the development of acute posttraumatic stress disorder-related symptoms after intensive care Crit Care Med 2001 16 573 580 10.1097/00003246-200103000-00019 11373423

[B21] Kress JP Gehlbach B Lacy M Pliskin N Pohlman AS Hall JB The long-term psychological effects of daily sedative interruption on critically ill patients Am J Resp Critic Care Med 2003 16 1457 1461 10.1164/rccm.200303-455OC 14525802

[B22] Richter JC Waydhas C Pajonk FG Incidence of posttraumatic stress disorder after prolonged surgical intensive care unit treatment Psychosomatics 2006 16 223 300 10.1176/appi.psy.47.3.223 16684939

[B23] Dowdy DW Dinglas V Mendez-Tellez PA Bienvenu OJ Sevransky J Dennison CR Shanholtz C Needham DM Intensive care unit hypoglycemia predicts depression during early recovery from acute lung injury Crit Care Med 2008 16 2726 2733 10.1097/CCM.0b013e31818781f5 18766087 PMC2605796

[B24] Dowdy DW Bienvenu OJ Dinglas VD Sevransky J Shanholtz C Needham DM Are intensive care factors associated with depressive symptoms six months after acute lung injury? Crit Care Med 2009 16 1702 1707 10.1097/CCM.0b013e31819fea55 19357507 PMC2769249

[B25] National Statistics National Statistics Socio-economic Classification (NS-SEC) 2010 London: National Statistics http://www.ons.gov.uk/ons/guide-method/classifications/current-standard-classifications/soc2010/soc2010-volume-3-ns-sec--rebased-on-soc2010--user-manual/index.html

[B26] Knaus WA Zimmerman JE Wagner DP Draper EA Lawrence DE APACHE - Acute Physiology and Chronic Health Evaluation: a physiologically based classification system Crit Care Med 1981 16 591 597 10.1097/00003246-198108000-00008 7261642

[B27] Keene AR Cullen DJ Therapeutic Intervention Scoring System: update Crit Care Med 1983 16 1 3 10.1097/00003246-198301000-00001 6848305

[B28] McNair DM Lorr M Droppelman LF *Manual for the Profile of Mood States* 1971 San Diego, CA: Educational and Industrial Testing Service

[B29] Broadbent E Petrie KJ Main J Weinman J The Brief Illness Perception Questionnaire J Psychosom Res 2006 16 631 637 10.1016/j.jpsychores.2005.10.020 16731240

[B30] Foa EB Cashman L Jaycox L Perry K The validation of a self-report measure of posttraumatic stress disorder: the Posttraumatic Diagnostic Scale Psychol Assessment 1997 16 445 451

[B31] Ehring T Kleim B Clark DM Foa EB Ehlers A Screening for posttraumatic stress disorder: what combination of symptoms predicts best? J Nerv Ment Dis 2007 16 1004 1012 10.1097/NMD.0b013e31815c1999 18091194

[B32] Radloff LS The CES-D scale: a self-report depression scale for research in the general population Appl Psychol Meas 1977 16 385 401 10.1177/014662167700100306

[B33] Weinert C Meller W Epidemiology of depression and antidepressant therapy after Acute Respiratory Failure Psychosomatics 2006 16 399 407 10.1176/appi.psy.47.5.399 16959928

[B34] Covic T Pallant JF Conaghan PG Tennant A A longitudinal evaluation of the Center for Epidemiologic Studies-Depression scale (CES-D) in a rheumatoid arthritis population using Rasch analysis Health Qual Life Outcomes 2007 16 41 10.1186/1477-7525-5-41 17629902 PMC1950699

[B35] Spielberger CD Gorsuch RL Lushene R Vagg PR Jacobs GA Manual for the State-Trait Anxiety Inventory 1983 Palo Alto, CA: Consulting Psychologists Press

[B36] Kindler C Harms C Amsler F Ihde-Scholl T Scheidegger D The visual analog scale allows effective measurement of preoperative anxiety and detection of patients' anesthetic concerns Anesthesia Anal 2000 16 706 712 10.1097/00000539-200003000-00036 10702461

[B37] Ware J Kosinski M Keller SD A 12-Item Short-Form Health Survey: construction of scales and preliminary tests of reliability and validity Med Care 1996 16 220 233 10.1097/00005650-199603000-00003 8628042

[B38] Hsieh FY Bloch DA Larsen MD A simple method of sample size calculation for linear and logistic regression Stat Med 1998 16 1623 1634 10.1002/(SICI)1097-0258(19980730)17:14<1623::AID-SIM871>3.0.CO;2-S 9699234

[B39] Brewin CR Dalgleish T Joseph S A dual representation theory of posttraumatic stress disorder Psychol Rev 1996 16 670 686 8888651 10.1037/0033-295x.103.4.670

[B40] Ely EW Shintani A Truman B Speroff T Gordon SM Harrell FE Jr Inouye SK Bernard GR Dittus RS Delirium as a predictor of mortality in mechanically ventilated patients in the intensive care unit JAMA 2004 16 1753 1762 10.1001/jama.291.14.1753 15082703

[B41] Kraemer HC Stice E Kazdin A Offord D Kupfer D How do risk factors work together? Mediators, moderators and independent, overlapping, and proxy risk factors Am J Psychiat 2001 16 848 856 11384888 10.1176/appi.ajp.158.6.848

[B42] Harvey AG Brewin CR Jones C Kopelman MD Coexistence of posttraumatic stress disorder and traumatic brain injury: towards a resolution of the paradox J Int Neuropsychol Soc 2003 16 663 676 12755178 10.1017/S1355617703940069

[B43] Longo LP Johnson B Addiction: Part I. Benzodiazepines - side effects, abuse risk and alternatives Am Fam Physician 2000 16 2121 2128 10779253

[B44] House A Stark D Anxiety in medical patients Br Med J 2002 16 207 209 10.1136/bmj.325.7357.207 12142312 PMC1123725

[B45] Schelling G The effect of stress doses of hydrocortisone during septic shock on posttraumatic stress disorder in survivors Biol Psychiat 2001 16 978 985 10.1016/S0006-3223(01)01270-7 11750894

[B46] Herridge MS Cheung AM Tansey CM Matte-Martyn A Diaz-Granados N Al-Saidi F Cooper AB Guest CB Mazer CD Mehta S Stewart TE Barr A Cook D Slutsky AS for the Canadian Critical Trials Group One-year outcomes in survivors of the acute respiratory distress syndrome N Eng J Med 2003 16 683 933 10.1056/NEJMoa022450 12594312

[B47] Welch CA Harrison DA Hutchings A Rowan K The association between deprivation and hospital mortality for admissions to critical care units in England J Crit Care 2010 16 382 390 10.1016/j.jcrc.2009.11.003 20074907

