# Supplementary material for: Investigating risk factors for psychological morbidity three months after intensive care: a prospective cohort study
Source: Crit Care. 2012 Oct 15;16(5):R192. doi: 10.1186/cc11677 (PMC3682294; doi:10.1186/cc11677)
Supplement: Additional file 1 — Tables showing full multivariable analyses. Tables showing the first stage of multivariable analyses for PTSD, depression and anxiety, and full multivariable analyses for physical and mental quality of life outcomes. [file cc11677-S1.DOC]

Additional File One

**Title: Tables showing full multivariable analyses**

This file includes tables showing the first stage of multivariable analyses for post-traumatic stress disorder (PTSD), depression and anxiety (tables S1 to S6). First-stage multivariable analyses were carried out only if more than two significant factors were identified in a group of risk factors (socio-demographic, clinical, psychological or chronic health) in univariable analyses. If few risk factors were found, the first stage of multivariable analysis was omitted. For this reason only the second stage of multivariable analysis was carried out for physical and mental quality of life outcomes (tables S7 and S8).

**Table S1 Regression: Clinical variables* and PTSD**

**(first-stage multivariable analysis)**

|  | Unstandardised  coefficient | p-  value |
| --- | --- | --- |
| Highest TISS score | 0.03 | 0.94 |
| Number of types of organ support received | -0.64 | 0.62 |
| Days of sedation | 0.70 | 0.04 |
| Benzodiazepines (yes/no) | 7.35 | 0.02 |
| Inotropes (yes/no) | 5.90 | 0.07 |
| Antipsychotics (yes/no) | 6.07 | 0.06 |
| Number of drug groups | -2.22 | 0.16 |
| C-reactive protein | 0.01 | 0.20 |
|  |  |  |

PTSD was measured using the Posttraumatic diagnostic scale (PDS,

0-51 scale)

*The eight strongest of the ten clinical variables that were found

significant (p<0.05) in univariable analyses

**Table S2 Regression: Acute ICU psychological factors***

**and PTSD (first-stage multivariable analysis)**

|  | Unstandardised coefficient | p-  value |
| --- | --- | --- |
| ICU mood | 0.22 | 0.08 |
| ICU stress | 0.12 | 0.36 |
| ICU amnesia | 1.13 | 0.61 |
| ICU intrusions | 5.36 | 0.02 |
| BIPQ timeline | 0.79 | 0.04 |
| BIPQ concern | 0.48 | 0.24 |
| BIPQ emotional rep | 0.10 | 0.79 |
|  |  |  |

PTSD was measured using the Posttraumatic Diagnostic Scale (0-51 scale)

*ICU psychological factors that were found significant (p<0.05) in

univariable analyses

**Table S3 Regression: chronic health factors* and PTSD**

**(first-stage multivariable analysis)**

|  |  | Unstandardised coefficient | p-  value |
| --- | --- | --- | --- |
|  | Psychological history (yes/no) | 9.42 | <0.01 |
|  | Alcohol use (yes/no) | 7.76 | 0.03 |
|  | Any past traumas  (yes/no) | 3.30 | 0.16 |
|  |  |  |  |

PTSD was measured using the Posttraumatic Diagnostic Scale (0-51 scale)

*chronic health factors that were found significant (p<0.05) in

univariable analyses

**Table S4 Regression: clinical factors* and depression**

**(first-stage multivariable analysis)**

|  |  | Unstandardised coefficient | p-  value |
| --- | --- | --- | --- |
|  | Primary body system | -0.56 | 0.35 |
|  | Length of stay in hospital | 0.07 | 0.12 |
|  | Post-hospital discharge | -0.57 | 0.64 |
|  | Benzodiazepines (yes/no) | 5.69 | 0.07 |
|  |  |  |  |

Depression was measured using the Center for Epidemiologic Studies Depression

Scale (0-60 scale)

*clinical factors that were found significant (p<0.05) in univariable analyses

**Table S5 Regression: Acute ICU psychological factors* and**

**depression (first-stage multivariable analysis)**

|  |  | Unstandardised coefficient | p-  value |
| --- | --- | --- | --- |
|  | BIPQ timeline | 0.55 | 0.27 |
|  | BIPQ concern | 0.67 | 0.21 |
|  | BIPQ emotional representation | 0.37 | 0.46 |
|  | ICU mood | 0.30 | 0.01 |
|  | ICU intrusions | 3.10 | 0.28 |
|  | ICU amnesia | 1.57 | 0.58 |
|  |  |  |  |

Depression was measured using the Center for Epidemiologic Studies

Depression Scale (0-60 scale)

*Acute ICU psychological factors that were found significant (p<0.05) in

univariable analyses

**Table S6 Regression: Acute ICU psychological factors* and**

**anxiety (first-stage multivariable analysis)**

|  |  | Unstandardised coefficient | p-  value |
| --- | --- | --- | --- |
|  | ICU mood | 0.29 | 0.02 |
|  | ICU intrusions | 2.03 | 0.50 |
|  | BIPQ timeline | 0.96 | 0.07 |
|  | BIPQ concern | 0.08 | 0.88 |
|  | BIPQ emotional representation | 0.72 | 0.20 |
|  |  |  |  |

Anxiety measured using the State-Trait Anxiety Inventory (0-80 scale)

*ICU psychological factors that were found significant (p<0.05) in

univariable analyses

**Table S7 Regression of mental quality of life and risk factors***

|  | *Socio-demographic*  *factors* | *Socio-demographic,*  *clinical, chronic physical*  *factors* | *Socio-demographic, clinical, chronic physical, acute ICU psychological factors,*  *psychological history* |
| --- | --- | --- | --- |
|  | Unstandardised p-  coefficient value | Unstandardised p-coefficient value | Unstandardised p-  coefficient value |
| NSSEC2*†* | -8.89 0.40 | -7.44 0.07 | -4.11 0.29 |
| NSSEC3 | -2.88 0.34 | -4.43 0.13 | -3.13 0.24 |
| NSSEC4 | -4.94 0.32 | -5.47 0.25 | -3.99 0.40 |
| NSSEC5 | -10.04 0.01 | -8.74 <0.01 | -9.92 <0.01 |
| NSSEC6 | -10.49 <0.05 | -11.23 <0.01 | -9.640 <0.01 |
| Inotropes |  | -4.21 0.05 | -2.28 0.27 |
| Chronic physical (yes/no) |  | -6.52 <0.01 | -3.48 <0.07 |
| ICU mood |  |  | -.29 <0.01 |
| BIPQ emotional representation |  |  | -.07 0.85 |
| Psychological history (y/n) |  |  | -3.65 0.19 |

Mental quality of life was measured using the SF-12 (mental component summary score)

*All risk factors that were found significant in univariable analyses.

*†*Variables SEC2-SEC6 are dummy variables representing differences between occupational categories within the

National Statistics Socio-economic classification (NS-SEC) [25]. In each dummy variable the numbered category is compared with the baseline of category 1. (NS-SEC categories are: 1. Professions/managerial 2. Intermediate professions

3. Self-employed 4. Technical/craft 5. Semi-routine/routine 6. Unclassified)

**Table S8 Regression of physical quality of life and risk factors***

|  | *Clinical factors and chronic physical illness* | *Clinical factors, chronic physical illness, illness perceptions* |
| --- | --- | --- |
|  | Unstandardised p-  Coefficient value | Unstandardised p-  Coefficient value |
| Anesthetics (yes/no) | 4.37 0.06 | 4.35 <0.05 |
| Steroids (yes/no) | 4.81 <0.05 | 4.69 <0.05 |
| Chronic physical illness (yes/no) | -2.68 0.22 | -.34 0.87 |
| BIPQ timeline |  | -1.28 <0.01 |
| BIPQ concern |  | -0.45 0.24 |

Physical quality of life was measured using the SF-12 (physical component summary score)

*All risk factors that were found significant in univariable analyses
